# Recent revelations and future directions using single-cell technologies in chronic lymphocytic leukemia

**DOI:** 10.3389/fonc.2023.1143811

**Published:** 2023-04-06

**Authors:** Blaž Oder, Anastasia Chatzidimitriou, Anton W. Langerak, Richard Rosenquist, Cecilia Österholm

**Affiliations:** ^1^ Department of Molecular Medicine and Surgery, Karolinska Institutet, Stockholm, Sweden; ^2^ Institute of Applied Biosciences, Centre for Research and Technology Hellas, Thessaloniki, Greece; ^3^ Department of Immunology, Erasmus MC, University Medical Center Rotterdam, Rotterdam, Netherlands; ^4^ Clinical Genetics, Karolinska University Hospital, Stockholm, Sweden

**Keywords:** single-cell sequencing, genomics, epigenomics, transcriptomics, immunogenetics, tumor microenvironment, chronic lymphocytic leukaemia

## Abstract

Chronic lymphocytic leukemia (CLL) is a clinically and biologically heterogeneous disease with varying outcomes. In the last decade, the application of next-generation sequencing technologies has allowed extensive mapping of disease-specific genomic, epigenomic, immunogenetic, and transcriptomic signatures linked to CLL pathogenesis. These technologies have improved our understanding of the impact of tumor heterogeneity and evolution on disease outcome, although they have mostly been performed on bulk preparations of nucleic acids. As a further development, new technologies have emerged in recent years that allow high-resolution mapping at the single-cell level. These include single-cell RNA sequencing for assessment of the transcriptome, both of leukemic and non-malignant cells in the tumor microenvironment; immunogenetic profiling of B and T cell receptor rearrangements; single-cell sequencing methods for investigation of methylation and chromatin accessibility across the genome; and targeted single-cell DNA sequencing for analysis of copy-number alterations and single nucleotide variants. In addition, concomitant profiling of cellular subpopulations, based on protein expression, can also be obtained by various antibody-based approaches. In this review, we discuss different single-cell sequencing technologies and how they have been applied so far to study CLL onset and progression, also in response to treatment. This latter aspect is particularly relevant considering that we are moving away from chemoimmunotherapy to targeted therapies, with a potentially distinct impact on clonal dynamics. We also discuss new possibilities, such as integrative multi-omics analysis, as well as inherent limitations of the different single-cell technologies, from sample preparation to data interpretation using available bioinformatic pipelines. Finally, we discuss future directions in this rapidly evolving field.

## 1 Introduction

Chronic lymphocytic leukemia (CLL) is characterized by an expansion of malignant CD5^+^/CD23^+^ B cells, often detected in the peripheral blood of asymptomatic patients (1). The median age at diagnosis is 72 years and men are afflicted more frequently than women. The disease course can range from indolent with a nearly normal life expectancy to aggressive with a poor response to treatment. Although well-established clinical staging systems (2, 3) remain instrumental for determining disease burden, they fail to identify early-stage patients prone to developing aggressive disease. Instead, molecular biomarkers that provide prognostic and/or predictive information have successively been identified. These include i) the immunoglobulin heavy variable (IGHV) gene somatic hypermutation (SHM) status, which divides patients into a poor-prognostic group with unmutated IGHV genes (U-CLL) or a favorable-prognostic group with mutated IGHV genes (M-CLL) (4, 5), and ii) the presence (or absence) of certain genomic lesions, such as deletions of 13q (35-45%), 11q (10-20%), and 17p (5-7%), and trisomy 12 (10-15%), as well as *TP53* mutations (1, 6, 7). These molecular tests are performed for all patients prior to the start of first-line treatment and at subsequent lines of treatment (except the IGHV gene SHM status that is stable throughout the disease course) (1, 8).

**Figure 1:**
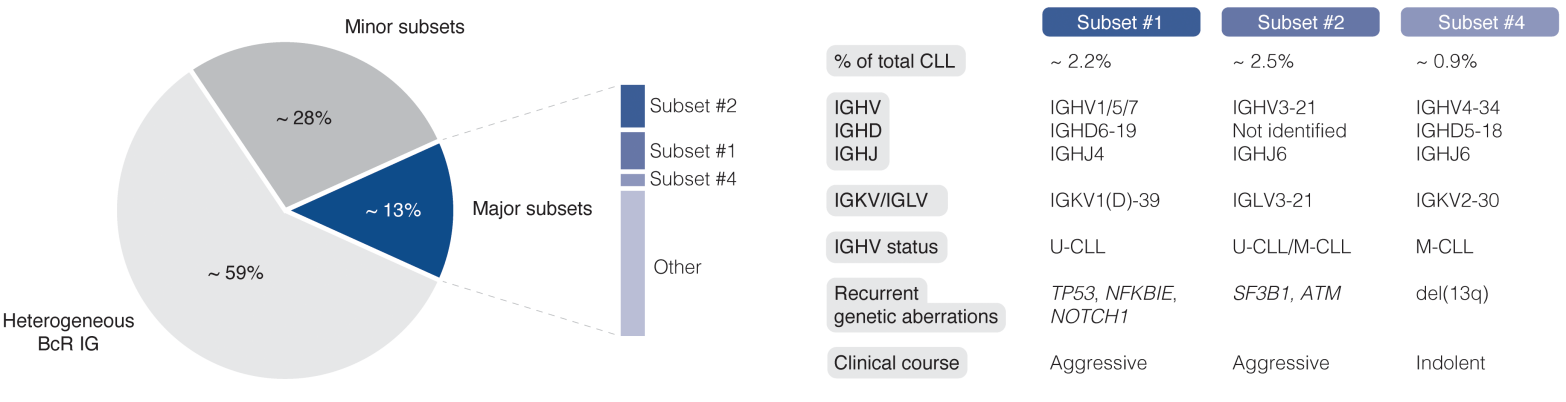
Clinicobiological profiles of major CLL subsets #1, #2, and #4. BcR IG, B cell receptor immunoglobulin; U-CLL, CLL with unmutated IGHV genes; M-CLL, CLL with mutated IGHV genes.

The easy access to tumor material from peripheral blood allows for advanced molecular studies of disease progress, from early, pre-cancerous monoclonal B cell lymphocytosis (MBL), to advanced stages of CLL, including Richter’s transformation (RT). With the introduction of next-generation sequencing (NGS) technologies more than 10 years ago, the genomic landscape of the different stages of CLL was rapidly uncovered. Today, recurrent genomic alterations have been described in >2000 genes, of which >200 genes have been identified as putative ‘drivers’. Of these, >25 genes are associated with clinically aggressive disease, including *ATM, BIRC3, EGR2, NFKBIE*, *NOTCH1*, *SF3B1*, and *TP53*, among others (9–14). Moreover, CLL is characterized by the expression of almost identical or ‘stereotyped’ B cell receptor immunoglobulins (BcR IGs) in more than 40% of patients (15). Notably, patients carrying stereotyped BcR IGs can be grouped into distinct subsets that display more similar molecular profiles and clinical outcomes than non-subset CLL patients (16, 17). For instance, patients in subset #1 (utilize IGHV1/5/7 clan I genes, U-CLL) and #2 (IGHV3-21/IGLV3-21, mixed SHM status) respond poorly to chemoimmunotherapy and have a dismal outcome, whereas subset #4 patients (IGHV4-34/IGKV2-30, M-CLL) show indolent disease courses and are rarely in need of treatment (**Figure 1**) (17, 18). Intriguingly, there is a striking enrichment of specific gene alterations in certain stereotyped subsets, for instance, *SF3B1* mutations in subset #2 and *TP53/NFKBIE/NOTCH1* aberrations in subset #1 (19). This biased acquisition of molecular lesions underscores the importance of both cell-intrinsic and cell-extrinsic factors in CLL pathobiology. In fact, besides the gradual accumulation of genomic lesions, the CLL clone is also dependent on active BcR signaling and interactions with the tumor microenvironment (TME) to promote clonal expansion. However, the TME plays a complex role in CLL pathobiology, where its constituents (including macrophages and their derivatives, mesenchymal stromal cells, and additional lymphocytes), participate in tumor-stimulating, reciprocal signaling, but also suppress anti-tumor immune surveillance mediated primarily by T cells (20–25).

**Figure 2:**
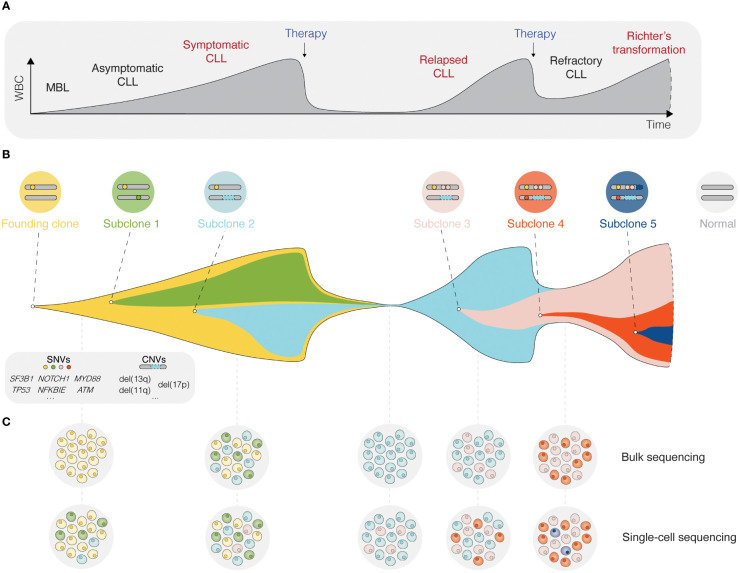
Single-cell sequencing deciphers cellular heterogeneity. **(A)** Schematic presentation of the different stages for a typical CLL patient, from the precursor condition monoclonal B cell lymphocytosis (MBL) to relapsed/refractory CLL, and in some cases Richter’s transformation. **(B)** The fish plot depicts tumor evolution in which multiple subclones exist within a leukemic cell population. While some clones are eradicated by therapy, others accumulate mutations that confer a clonal advantage. **(C)** The depth of bulk sequencing primarily allows detection of dominant subclones only, whereas single-cell analyses have the potential to detect all subclones, including minor ones as soon as they arise. WBC, white blood count; MBL, monoclonal B cell lymphocytosis; SNV, single nucleotide variant; CNV, copy-number variant.

Inhibition of BcR signaling or intrinsic apoptotic pathways by contemporary targeted therapies has shown significant clinical efficacy and prolonged progression-free and overall survival in poor-risk patients with CLL carrying *TP53* aberrations or unmutated IGHV genes (26–29). Nevertheless, the evolution and selection of therapy-resistant subclones may occur during and/or after targeted treatment and lead to disease relapse (**Figure 2**) (30). Notably, even minor alterations at the subclonal level (e.g., *BTK* and *PLCG2* mutations) are sufficient to drive treatment resistance (**Figure 2**) (31, 32). Deciphering the clonal evolution of CLL cells under treatment-induced selection pressure is thus critical for a better understanding of resistance mechanisms and the identification of additional predictive biomarkers.

The advent of single-cell sequencing technologies has enabled unprecedented dissection of the intraclonal molecular landscape, further linking genotypes and phenotypes to specific CLL cell subpopulations. In this review, we outline how various single-cell sequencing approaches can be used to unravel intratumoral heterogeneity and track clonal evolution in distinct phases of the disease (**Figure 2**), but also the contribution of the TME. We highlight the limitations of single-cell technologies and discuss new directions, such as spatial omics and integrated, multi-omics single-cell analysis.

## 2 Deciphering clonal heterogeneity and evolution in CLL

As mentioned, CLL is notable for significant genetic diversification and clonal evolution, both during disease progression and upon therapeutic interventions (10, 33, 34). The occurrence of clonal heterogeneity became evident in the 1980-90s, initially by applying traditional cytogenetic techniques, such as chromosome banding analysis and fluorescence *in situ* hybridization (FISH) (35–37). With the development of new technologies, including array- and NGS-based approaches (38, 39), the resolution of detection has increased continuously, enabling to follow clonal dynamics albeit still at a ‘bulk’ level (40). Frequently used NGS methods include whole-genome sequencing (WGS), whole-exome sequencing (WES), RNA-sequencing (RNA-seq), and analyses of the epigenome, such as methylation profiling and assay for transposase-accessible chromatin with sequencing (ATAC-seq). Using high-resolution genomic technologies, it has been possible to discern early versus late molecular events during CLL pathogenesis (**Figure 2**). Consequently, we now know that a few genomic aberrations represent early clonal events (e.g., trisomy 12, *MYD88* mutations, and del(13q)), whereas most alterations are present at the subclonal level (10, 12, 41). These technologies have been instrumental in identifying important CLL-specific genomic, epigenomic, and transcriptomic features linked to key dysregulated signaling pathways and cellular processes and have also enabled a multi-layered integrative portrayal of CLL and the discovery of novel subgroups. For instance, in three recent studies, novel patient clusters with distinct clinicobiological features and outcomes were identified using multi-omics approaches, including proteogenomics (12, 14, 42). Additionally, targeted deep-sequencing has become a powerful tool for further in-depth molecular characterization of CLL, allowing for the discovery of previously undetected, smaller-sized lesions that occur at low frequencies. As a concrete example, minor *TP53*-mutated subclones, undetected by Sanger sequencing but identified by ultra-deep NGS, have been shown to influence clinical outcomes negatively, at least in patients treated with chemoimmunotherapy (43, 44). Furthermore, emerging treatment resistance related to targeted therapy, such as *BTK, PLCG2*, and *BCL2* mutations, can be detected by deep-sequencing or droplet digital PCR (ddPCR) of hotspot positions (45–47).

While these new technologies have advanced the field in terms of deciphering clonal dynamics and treatment resistance in CLL, there are evident limitations when analyzing bulk nucleic acids and proteins from a heterogeneous leukemic sample. Instead, single-cell technologies have opened new possibilities for in-depth studies of clonal diversity in malignant diseases, including CLL.

### 2.1 Single-cell sequencing technologies

**Figure 3:**
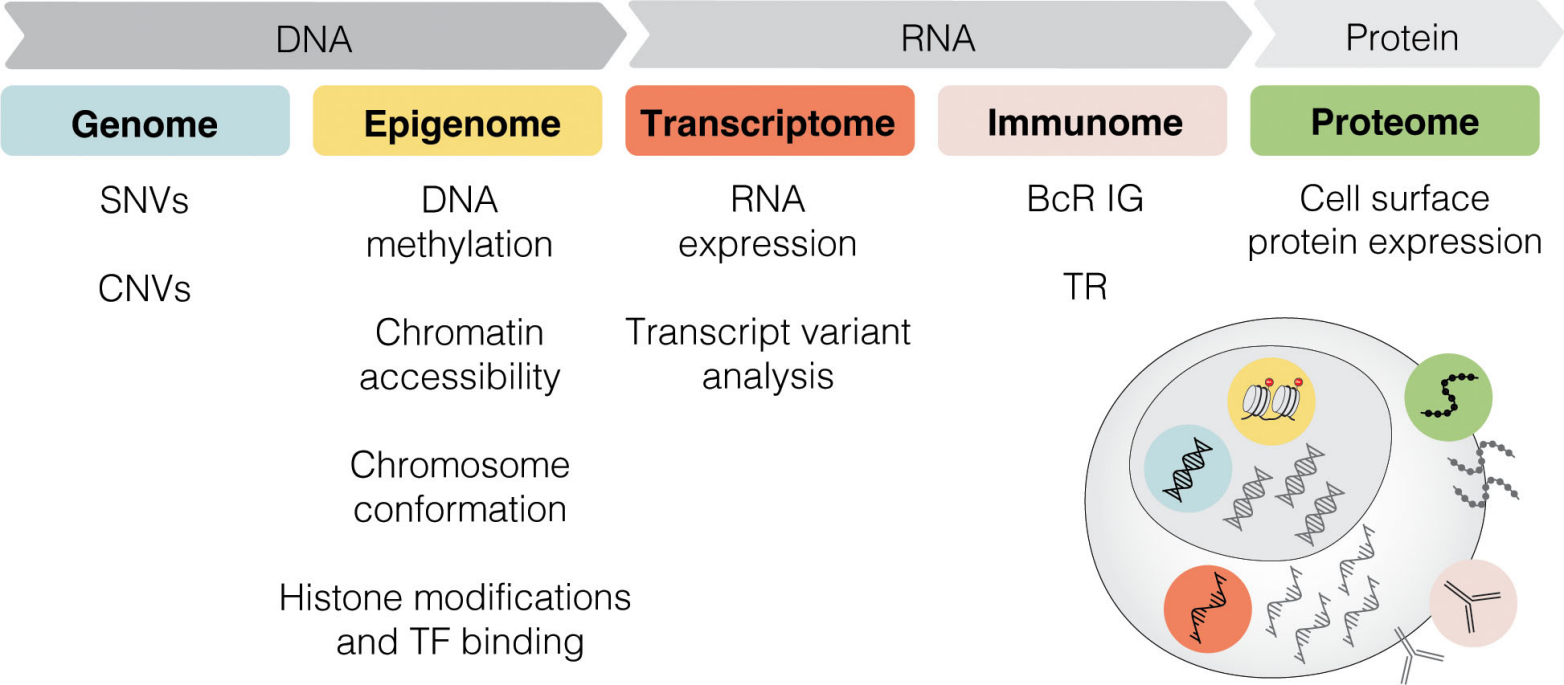
Multi-modal profiling. SCS methods can detect alterations across multiple modalities, including genome, epigenome, transcriptome, immunome, and proteome. SNV, single nucleotide variant; CNV, copy-number variant; TF, transcription factor; BcR IG, B cell receptor immunoglobulin; TR, T cell receptor.

**Figure 4:**
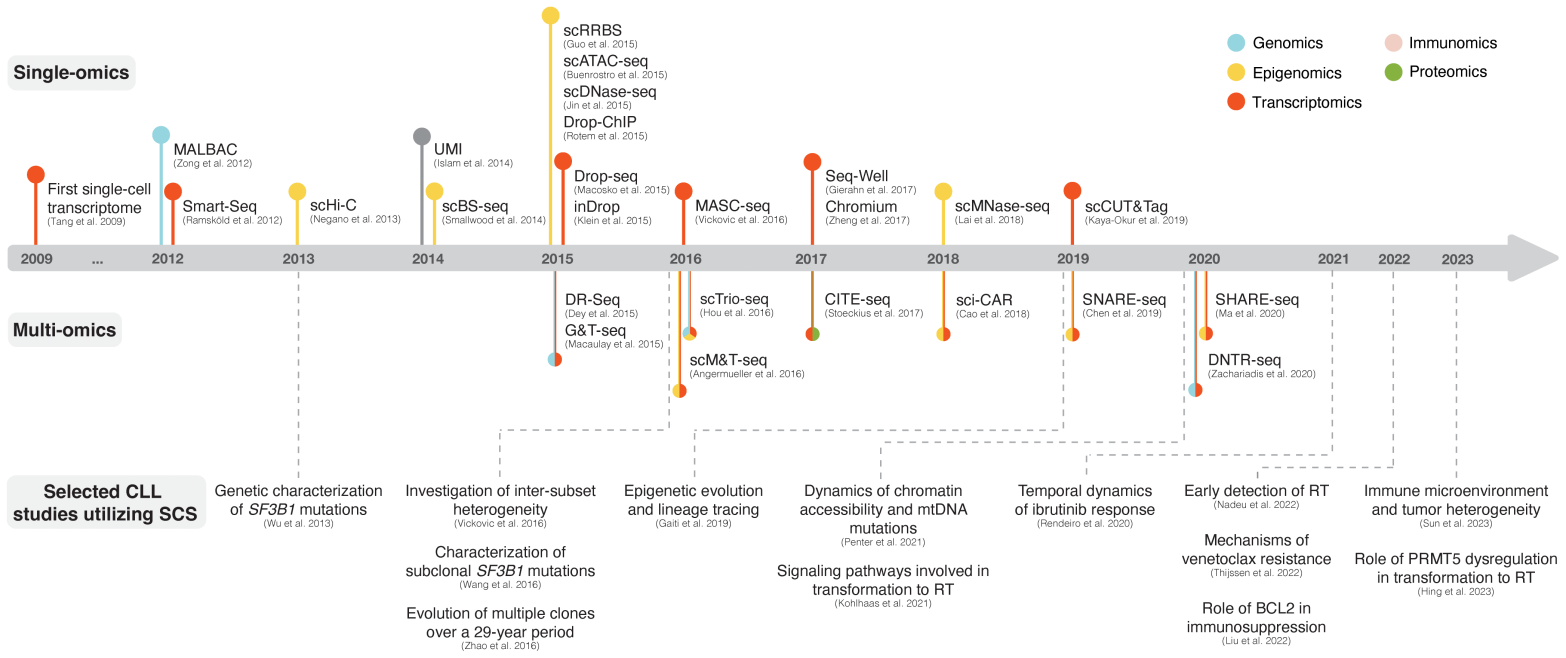
The advent of SCS technologies with selected examples of CLL studies. The respective panels above and below the timeline showcase various single-omics and multi-omics technologies that have been developed over the last decade. Selected CLL studies where SCS was employed are exemplified in the lower part of the figure.

Single-cell sequencing (SCS) technologies, particularly when employed in combination and on longitudinal sets of samples, enable the dissection of subclonal composition and evolutionary dynamics, both in the context of disease progression and response to treatment (48). Moreover, they hold the potential to reveal druggable signaling pathways and mechanisms contributing to treatment resistance. In the following sections, we introduce various SCS technologies and their utility across different modalities (the latter summarized in **Figure 3**) and discuss their contribution to the understanding of CLL pathogenesis and potential clinical implications. We also provide a timeline of SCS from a technology development aspect, which includes milestones of CLL single-cell research (**Figure 4**).

#### 2.1.1 Single-cell transcriptomics

One of the first SCS applications was single-cell RNA sequencing (scRNA-seq), which, owing to its ability to assess transcriptomes of individual cells, marked a paradigm shift in cancer research (49–51). The initial scRNA-seq methods, such as Smart-Seq, were low-throughput and relied on sorting single cells into multi-well plates and sequencing of full-length cDNA libraries obtained through whole-transcriptome amplification (WTA) with oligo(dT) priming and template switching (**Figure 4**) (52, 53). The introduction of unique molecular identifiers (UMIs) facilitated more reliable and absolute quantitation of mRNA molecules with nearly eliminated amplification bias (54). Later, several microwell- and droplet-based scRNA-seq methods based on 3´ cDNA libraries (and less often 5´ cDNA libraries, which are designed to be combined with profiling of cell surface proteins and/or immune repertoire) were developed, such as Seq-Well (55), Drop-seq (56), inDrop (57), and Chromium (**Figure 4**) (58). These platforms overcame previous limitations of only sequencing tens to hundreds of cells by enabling sequencing of thousands of cells at a time as well as unlocking the potential to capture transcriptomes from heterogeneous cell populations more accurately, but at less comprehensive coverage. For a more detailed overview of scRNA-seq methods, we refer to several extensive review articles (49–51, 59).

In CLL, scRNA-seq has helped to resolve the transcriptomic changes and alternative splicing effects of *SF3B1* mutations, a common subclonal event associated with clinically aggressive disease (**Figure 4**) (41). In a study by Wang et al., single cells carrying an *SF3B1* mutation possessed significant changes associated with multiple cellular functions, including apoptosis (upregulation of *BIRC3*, *BCL2*, and *KLH21*), DNA damage and cell cycle (increase of *KLF8*, *ATM*, *CDKN2A*, and *CCND1*), telomere maintenance (upregulation of *TERC* and *TERT*), and NOTCH signaling (downregulation of *DTX1* and altered splicing of *DVL2*) (60). Through this study, scRNA-seq was demonstrated to be applicable for in-depth investigation of subclonal events, whose effects could be missed when assessed by ‘bulk’ sequencing approaches. In a previous study, we used MASC-seq, a method based on single-cell microarray capture of mRNA, to show differences in transcriptional expression patterns among CLL cells from patients classified into distinct stereotyped subsets (i.e., subsets #1, #2, and #4) (**Figure 4**). Using this approach, we observed major and minor clusters of CLL cells with unique expression signatures in each case (61). Another comprehensive and high-resolution single-cell multi-omics study, in which combined scRNA-seq and ‘bulk’ ATAC-seq were performed to address ibrutinib treatment response, discovered a tightly regulated ibrutinib-induced signaling program of CLL cells (**Figure 4**). Initially, reduced expression of *BTK*, *CD52* (a CLL disease activity marker), and *CD27* (a memory B cell marker) and diminished chromatin accessibility at NF-κB binding sites were observed, followed by a rapid decrease in the activity of B cell lineage-defining transcription factors (EBF1, FOXM1, IRF4, PAX5, and PU.1), loss of CLL cell identity, and acquisition of a quiescent-like gene signature (upregulation of *CXCR4*, *ZFP36L2*, and *HMGB2)* (62).

#### 2.1.2 Single-cell genomics

In contrast to scRNA-seq approaches, the development of single-cell DNA sequencing (scDNA-seq) methods has proven to be more difficult since a single cell contains only two copies of genomic DNA. Multiple methods for uniform whole-genome amplification (WGA), such as multiple annealing and looping-based amplification cycles (MALBAC), have been developed and enabled single-cell WGS (scWGS) although at a modest throughput (63) (**Figure 4**). To facilitate charting of the most prevalent gene mutations during clonal evolution, also in the context of CLL (64), high-throughput targeted DNA sequencing using disease-specific gene panels has become available for single-cell analysis. Furthermore, several approaches have been developed for performing simultaneous scRNA-seq and scDNA-seq on the same cell, such as DR-Seq (65) and G&T-seq (66), both of which are based on whole cell lysis and subsequent separation of poly-adenylated RNA from genomic DNA, and direct nuclear tagmentation and RNA-seq (DNTR-seq) (67), in which nucleus and cytosol are physically separated beforehand (**Figure 4**).

Early efforts to use scDNA-seq in CLL research focused on evaluating *SF3B1* mutations, which had long been assumed to be heterozygous in CLL (68), owing to their typical allelic burden of <50% (**Figure 4**). Using targeted scDNA-seq, single CLL cells indeed demonstrated heterozygous genotypes, however, a novel subpopulation with homozygous *SF3B1* mutant genotype was discovered, supporting a subclonal evolutionary pathway of *SF3B1* mutations in CLL (69). This study illustrates the applicability of scDNA-seq in detecting subclonal populations not possible to unmask by ‘bulk’ approaches. Another study, in which scWGS was combined with scRNA-seq and performed on a longitudinal set of samples collected from one patient over a 29-year disease course, demonstrated the power of single-cell approaches for reconstructing cancer evolution based on CNVs and changes in gene expression (**Figure 4**). Clonal selection in response to treatment was manifested by the disappearance of certain populations and the emergence of a clone with novel CNVs, whereas disease progression was reflected by dynamic transcriptome changes, including upregulation of transcription factors involved in stem cell and cell cycle regulation (*KLF4*, *KLF6*, and *CDKN1A)*, MYD88 signaling (*FOS*, *JUN*, and *NFKBIA*) and downstream of BcR signaling (*REL*, *CDKN1A*, and *NFKBIA*) (70). In a recent study, scDNA-seq of 32 genes, scRNA-seq, and high-throughput immunogenetic analysis were performed on longitudinal samples in patients developing RT, revealing that micro subclones could be identified already at CLL diagnosis up to 19 years before transformation (**Figure 4**) (64).

#### 2.1.3 Single-cell epigenomics

With the advent of scDNA-seq, SCS approaches for capturing epigenomic alterations have flourished as well and today allow the assessment of methylation dynamics and programs for transcriptional regulation. These technologies were adapted from methods originally applied on bulk nucleic acids and vary based on the epigenomic modality being assayed. Frequently used methods include i) investigation of methylome by single-cell DNA methylation sequencing (scDNAme-seq) approaches that entail bisulfite conversion of genomic DNA, such as scRRBS (71) and scBS-seq (72), ii) analysis of chromatin accessibility by single-cell ATAC-seq (scATAC-seq) (73), single-cell DNase sequencing (scDNase-seq) (74), and single-cell micrococcal nuclease sequencing (scMNase-seq) (75), iii) exploration of the spatial genome organization and chromatin interactions using chromosome conformation capture, such as single-cell Hi-C (76), and iv) interrogation of histone modifications and transcription factor binding by single-cell chromatin immunoprecipitation followed by sequencing (scChIP-seq), such as Drop-ChIP (77), and single-cell cleavage under targets and tagmentation (scCUT&Tag) (**Figure 4**) (78–80). Furthermore, multi-omics approaches have been developed for combined capturing of methylome and transcriptome, such as scM&T-seq (81), chromatin accessibility and transcriptome, such as SNARE-seq (82) and SHARE-seq (83), and even combinations of the methylome, genome, and transcriptome, such as scTrio-seq (**Figure 4**) (84).

While epigenetic studies on bulk material have provided important clues as to the cellular origin of CLL and also identified subtypes with distinct DNA methylation profiles and outcome (85, 86), single-cell epigenomics paves the way for investigation of epigenetic programming and transcriptional regulatory networks in evolving clones. A recent study, in which scDNAme-seq was combined with scRNA-seq, showed that an increased proliferative capacity of CLL cells was reflected in consistently increased epimutation rates with minimal cell-to-cell variability in contrast to healthy B cells (**Figure 4**) (87). Furthermore, the authors demonstrated that mapping of epimutations can be used as a means for subclone lineage reconstruction and tracing, consistent with previous reports (85, 86). CLL cells with elevated epimutation rates exhibited higher gene expression heterogeneity (also known as transcriptional entropy), consistent with transcriptional dysregulation. Additionally, the investigators demonstrated the enrichment of low epimutation rates in gene promoters for binding motifs of transcription factors with established roles in CLL progression (*NFKB1* and *MYBL1*), and enhancers in proximity to genes implicated in lymphoproliferation (*NOTCH1*, *NFATC1*, and *FOXC1*) and key CLL signaling pathways (e.g, Wnt and MAPK) (87). Another study addressing temporal clonal dynamics employed mitochondrial scATAC-seq and scRNA-seq and revealed that naturally occurring mutations in mitochondrial DNA (mtDNA) could be utilized as biomarkers to distinguish between CLL cell subpopulations with distinct functional states (**Figure 4**) (88). The presence of mtDNA mutations closely mirrored the disease history and reflected the acquisition of CNVs as well as changes in chromatin accessibility and gene expression, allowing for tracking of existing clones and assessing the emergence of divergent subclones with varying fitness over time, particularly in response to therapy (88).

#### 2.1.4 Single-cell proteomics

The advancement of proteogenomics has allowed for the study of relationships between genetic/transcriptional features and protein expression at ‘bulk’ level, also in CLL (42, 89, 90). While various single-cell platforms are now commercially available for sequencing nucleic acids, whole-proteome analysis at the single-cell level is still under development. Protein-related difficulties include the wide range of post-translational modifications and the inability of peptides to be amplified. As a result, current efforts are primarily focused on increasingg the signal-to-noise ratio by reducing sample processing volumes and ion contamination (91). Until standardized mass spectrometry-based single-cell proteomics is available, the field is dominated by other techniques. Cellular indexing of transcriptomes and epitopes by sequencing (CITE-seq) (92), which uses an antibody-oligonucleotide conjugate-based approach, enables multiplex quantitative profiling of cell surface proteins and has recently been applied in phenotyping of CLL cells for investigating mechanisms of venetoclax resistance, based on short- and long-read scRNA-seq (**Figure 4**). This study demonstrated a high plasticity of CLL cells in their ability to evade apoptosis upon venetoclax treatment, likely through NF-κB-induced upregulation of the pro-survival protein MCL1 (93).

#### 2.1.5 Single-cell immunomics

Alterations of immunogenetic features constitute a central aspect of clonal evolution in CLL. Using low-throughput sequencing approaches, we have previously demonstrated the occurrence of intraclonal diversification based on SHM patterns of the clonotypic IGH/IGK/IGL gene rearrangements, particularly in subset #4 patients (94, 95). Today, standardized protocols for NGS-based IGH/IGK/IGL sequencing have been developed and enable an in-depth analysis of the clonal composition and dynamics over time (96, 97). In fact, a recent study focusing on stereotyped subset #2 and #169 (a satellite subset to subset #2) demonstrated shared SHM patterns in both subsets at either clonal or subclonal level, reflecting ongoing intraclonal diversification compatible with a branched evolution (98). The recently developed strategy to combine immunomics (99) with global transcriptomics in individual cells, allows for B cell and/or T cell clonotypes to be linked to gene expression signatures (100). Collectively, these technologies facilitate in-depth analysis of immunogenetic features (e.g., ongoing SHM and class-switching) at single-cell level and the discovery of clone-specific phenotypes, which may in turn expedite the identification of therapeutic targets and resistance markers.

### 2.2 The role of the microenvironment

Major sites for CLL cell propagation are the primary and secondary lymphoid tissues, such as bone marrow (BM) and lymph nodes (LN). The TME, populated also by non-tumor leukocytes and mesenchymal stromal cells, provides stimulatory and anti-apoptotic signals to the malignant clones (21, 101–103) and interplay with tumor cell-intrinsic factors to promote resistance (104). Within the TME, the T cell population is of particular interest, due to its tumor-restricting abilities. In parallel to the biased IG gene repertoire in CLL in general and in patients with stereotyped BcR IGs in particular, the T cell receptor (TR) repertoire is oligoclonal and skewed in terms of the TR beta (TRBV) gene usage, as previously demonstrated by both low-throughput sequencing analysis and NGS (105–107). Furthermore, similar to other malignancies, T cell exhaustion sustained by continuous antigen exposure and manifested as altered chemokine secretion, reversed ratios of CD4^+^/CD8^+^ cells, altered CD4^+^ cell helper function, and diminished CD8^+^ cell cytotoxicity is a common feature of CLL (22, 23, 108, 109).

Concurrent single-cell profiling of TR gene rearrangements and global transcriptomes aids in the investigation of T cell clonality while also elucidating phenotypes and immuno-responsiveness (99, 110). To map transcriptional profiles of T cells in patients with CLL, scRNA-seq was applied on pre-sorted T cells with or without *BCL2* expression. Increased BCL2 levels were suggested to be a marker of T cell dysfunction, and treatment with venetoclax, a BCL2 inhibitor, was able to restore functional T cell immunity by removing BCL2-positive T cells (111). In another study addressing the role of IL-10 receptor signaling in CD8^+^ T cell exhaustion based on the Eμ-TCL1 mice model, scRNA-seq aided in identifying transcriptional profiles of CD8^+^ T cells associated with different surface levels of PD1, indicative of an exhausted versus immuno-responsive phenotype (112).

While the abundance of CLL cells in the peripheral blood allows for easy access to tumor material, peripheral blood mononuclear cells (PBMCs) do not represent the active, proliferating tumor population to a large extent. By performing ‘bulk’ and scRNA-seq on CLL cells isolated from LNs as well as peripheral blood, Sun et al. determined the fraction of activated cells in the LNs to a few percent of the total CLL cell count in the LN compartment. This corroborates previous estimations from *in vivo* labeling of CLL cells using deuterated water (113). A portion of the proliferating cells was also shown to proceed in a unidirectional fashion with mitosis followed by activation and subsequently by quiescence (114). Another study utilizing scRNA-seq on spleen- and LN-derived CLL cells from Eμ-TCL1^Akt-C^ mice in a model of RT pointed to the importance of sustained Akt signaling for maintaining a pro-proliferative and anti-apoptotic microenvironment through aberrant NOTCH1 activation (115). scRNA-seq was also instrumental in identifying the epigenetic modifier PRMT5 as a potential mediator of RT, in patient tissues as well as in an experimental model employing Eμ-PRMT5/TCL1 mice (116).

While SCS on extracted cells give valuable insight regarding the different disease-driving compartments, attaining information based on spatial context necessitates a preserved histological architecture. The critical role of the stroma and the geographic location of different stromal cell populations in relation to tumor cells (i.e., tumor/stroma boundaries) have been illustrated by spatial multi-omics in solid tumors (117). This pertains not only to direct cell/cell communication but also the composition dynamics of the extracellular matrix and the complex interplay with its producers. Over the last years, the technology has shifted from an initial oligo(dT)-based strategy relying on high-quality, fresh-frozen tissue (118), to gene-specific capture probes with rapidly increasing transcriptome coverage, which makes the currently available platforms for spatial transcriptomics and proteomics suitable for utilization of archival, paraffin-embedded and cryopreserved material containing RNA of compromised integrity (119, 120). In the case of CLL, increased access to detailed information about properties of rapidly dividing tumor cells and accessory cells within the TME will be instrumental to develop new strategies to effectively target the site of birth for CLL cells. Additionally, it will allow for a more meticulous exploration of mechanisms leading to loss of homing and lymphocytosis upon treatment with BTK inhibitors.

## 3 Discussion

**Figure 5:**
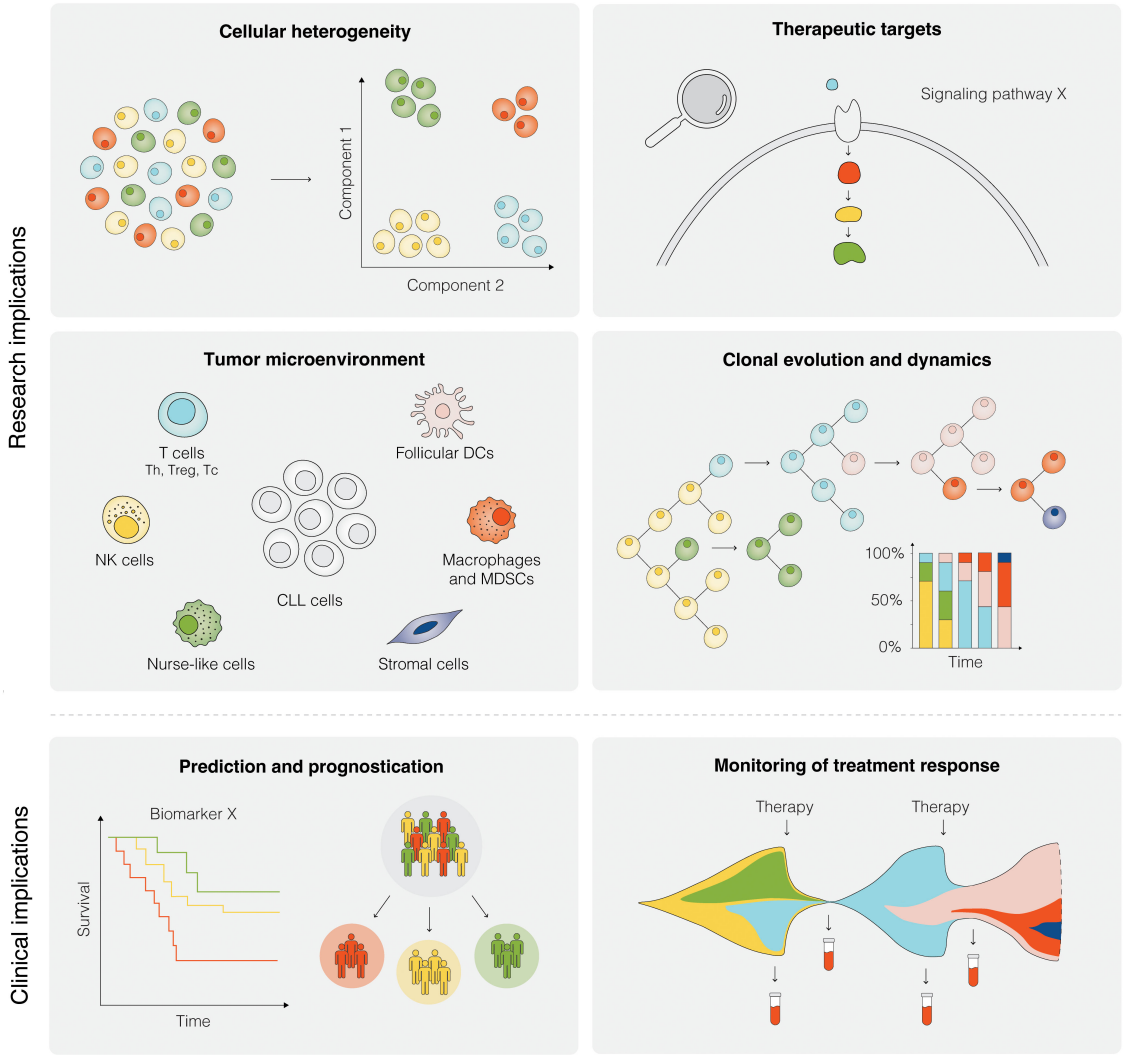
Research and clinical implications of SCS in CLL. Research implications of SCS include studies of cellular heterogeneity, identification of potential therapeutic targets, investigation of the TME, and analyses of clonal evolution and dynamics, among others. Clinical implications of SCS are still remote, but could include the prediction of therapeutic intervention, prognostication of clinical outcome, and therapy monitoring.

So far, SCS technologies have provided a wide range of options for identifying molecular features at the transcriptomic, genomic, and epigenomic level. In the case of CLL, these methods facilitate the construction of a comprehensive and detailed map of clonal heterogeneity and evolution over time and in response to, above all, targeted treatment (**Figure 5**). An illustrative example presented above is the ability to identify subclones implicated in RT, which may be present at the time of CLL diagnosis and for up to two decades before clinical manifestation (64). Other important contributions empowered by SCS and which will be valuable for elucidating additional mechanisms behind drug resistance and therapy-induced biological adaptation to BTK and BCL2 inhibitors, include lineage tree reconstruction and identification of key signaling pathways and early markers of progression. Using approaches like this, SCS will help us to better understand which patients are more likely to experience an aggressive disease course, regardless of disease burden at the time of diagnosis. It will also allow us to decipher the subclonal composition of less well studied subgroups of CLL, such as subsets expressing stereotyped BcR IGs, associated with distinct outcomes (**Figure 1**) (17). Here, an advantage pertains to the ability to perform concomitant single-cell analysis of the transcriptome and expressed IG genes. Additionally, scRNA-seq enables the generation of transcriptional profiles from other accessory cells obtained from PBMCs and lymphoid tissues without the bias implied by prior selection/sorting. Combined with high-resolution spatial omics technologies, this allows for a detailed characterization of the TME with emphasis on pro-proliferative and immunoregulatory properties, and will further aid to identify mechanisms of resistance to contemporary therapies. Some limitations and challenges should be considered when designing studies, preparing samples, and analyzing and interpreting data.

Theoretically, SCS has the potential to detect and in detail investigate minor clones and accessory cells with rare genotypes and/or phenotypes. This, however, necessitates the sequencing of a considerable number of cells, using a sufficient sequencing depth. Therefore, under current circumstances, SCS is less suitable for finding very small subclones or for detection of measurable residual disease due to the current high costs of performing these assays as well as the high resolution and robust output obtained with other established and clinically validated protocols (ddPCR and ultra-deep NGS) on bulk nucleic acids (121–123). Nonetheless, with the anticipated decrease in the cost of sequencing in the coming years, this will enable analyses of a greater number of cells.

Reduced sample viability of cryopreserved cells, due to biological variation or extrinsic factors, may also present a problem as it impacts data quality and reproducibility. A recent study by Massoni-Badosa et al. found that extended storage of PBMCs prior to sample preparation and scRNA-seq had a significant impact on gene expression profiles of PBMCs from healthy subjects and CLL patients, even though RNA integrity was preserved during longer storage times. The effect was most noticeable in global gene expression and, to a lesser extent, open chromatin patterns, as measured by scATAC-seq (124). Because simultaneous sampling and the use of freshly harvested samples are generally not possible, the importance of standardized protocols across studies and collaborating centers cannot be overstated (125). Although the issue of initial PBMC storage and processing remains, the recently emerging possibility of using paraformaldehyde-fixed cells for scRNA-seq avoids the challenge of maintaining high cell viability through cryopreservation and transportation (126, 127). Furthermore, if multi-omics is used, different applications may necessitate different sample preparation methods, which in turn requires careful coordination.

SCS technologies have led to a data revolution in CLL research, which inevitably brings challenges also when analyzing and interpreting such data. A major issue usually concerns the lack of appropriate references for the identification of different cell types from heterogeneous samples, although this is not aggravated in CLL where the majority of cells in PBMC samples are leukemic. While several annotation tools based on the expression of cell-type-specific markers have been developed for scRNA-seq, these may not be reliable for the discovery of rare, uncharacterized cell populations or small leukemic subpopulations in heterogeneous samples (128–130). Since such tools are not yet available for most other SCS platforms, cell type assignment is often performed manually using clustering and dimensionality reduction methods, which limits the reproducibility of the results. False cell type assignment can thus have an impact on downstream analyses such as differential gene expression and lineage tree reconstruction. Although potentially challenging, validation by independent methodologies, such as PCR-based methods, perturbation experiments and flow cytometry, is therefore necessary to ascertain the accuracy and reliability of obtained results. Sparse transcriptomic profiles present another frequent challenge when analyzing scRNA-seq data, and depends not only on the initial relative mRNA abundance but also on technical constraints related to amplification bias, libraries with uneven coverage, and sequencing depth (130–132). Therefore, cell-to-cell variability within the same population must be considered, and absent gene expression should be interpreted cautiously. As there is evidence of aberrant RNA splicing induced by *SF3B1* mutations, as well as an increasing indication of the involvement of long non-coding RNAs and microRNAs in the CLL pathogenesis (13, 133–135), investigating these effects at single-cell resolution has become of considerable interest. However, such analyses of alternative splicing and non-coding RNAs are limited because most scRNA-seq platforms rely on 3’ and 5’ libraries, which represent merely 3´or 5´parts of transcripts. Despite this, 3´and 5´-based scRNA-seq is advantageous due to a reduced technical noise compared to WTA, and to cost-effectiveness as it requires less sequencing depth to obtain sufficient coverage of gene expression. Nonetheless, for the aforementioned analyses single-cell long-read RNA-seq approaches are gaining momentum and have already demonstrated higher proportions of novel transcripts in CLL (136).

The primary difficulty of scDNA-seq concerns WGA, which can introduce amplification errors and bias towards imbalanced proportions of alleles or even drop out of variant alleles, resulting in unreliable variant detection that consequently hinders characterization of intertumoral heterogeneity and reconstruction of evolutionary history (130). To address uneven genome coverage and challenging variant calling, targeted scDNA-seq, in which only regions of interest are selectively amplified, is gaining popularity and is now commercially available also for many hematological malignancies, including CLL (137, 138). Similarly, analyses of other SCS data may be biased due to inadequate sequencing coverage and depth.

To allow for more systematic and comprehensive studies of CLL pathobiology, in particular clinically aggressive subgroups, integration of various omics is necessary. A new subgroup of patients with aggressive disease (20% of patients) was recently identified by using proteogenomics at ‘bulk’ level, which could not be identified by genomic analyses alone, emphasizing the value of superimposing and integrating different layers of information (42). Additionally, extended proteomics that accommodates analyses of diverse post-translational modifications, such as phosphorylation and glycosylation, will likely contribute to refining signatures of aggressive and/or therapy-resistant clones, when performed at the single-cell level.

Despite the recent development of rigorous statistical and computational frameworks, such as multi-omics factor analysis (MOFA) (139, 140), omics integration remains a challenge (128, 141). As multi-omics approaches that allow for simultaneous capture of different omics in the same cells are on the rise, data integration may become easier, however, other challenges, such as accounting for dependencies among the measurement types, may emerge. A related data analysis issue concerns comparisons across samples and multiple batches, for which multiple bioinformatics tools have been developed as well (142–144).

Since SCS is still a rapidly evolving field, no methods and bioinformatics pipelines regarded as ‘gold standard’ currently exist for data analysis, leaving researchers to rely on various options, and base their selection on availability, price, labor intensity, method complexity, and expertise in bioinformatics. Considering that each method has its advantages and disadvantages, the ‘right’ approach should be carefully selected based on the desired application.

## 4 Conclusions

The SCS methods described above have extended the range of possibilities for identifying novel signatures and recurrent markers of clonal evolution and treatment resistance in CLL and have also enabled a detailed deciphering of molecular events in anatomical sites that constitute the epicenters of disease progression, more particularly the LNs (**Figure 5**). While the technologies are under constant development, the current clinical utility of SCS methods *per se* is still in its infancy. Potential future clinical applications include assessment of clonal and microenvironmental composition prior to and during targeted therapy as well as monitoring of treatment response (**Figure 5**). Nonetheless, through the possibility to capture the genome, epigenome, transcriptome, immunome, and to a limited extent also the proteome in individual cells, SCS signifies a paradigm shift in CLL research. This detailed dissection of the disease at the cellular level will have implications for patient stratification and management in terms of diagnostics, prognostics and tailoring of treatment. As discussed here, SCS poses several challenges; thus, the different aspects of a study, including study design, sample preparation, data analysis, and interpretation should be considered using an integrated approach.

## Author contributions

BO designed the review layout, wrote the paper and designed the figures. AC and AL wrote the paper. RR and CÖ designed the review layout and wrote the paper. All authors contributed to the article and approved the submitted version.
